# Impact of Reconstruction Algorithms on CT Radiomic Features of Pulmonary Tumors: Analysis of Intra- and Inter-Reader Variability and Inter-Reconstruction Algorithm Variability

**DOI:** 10.1371/journal.pone.0164924

**Published:** 2016-10-14

**Authors:** Hyungjin Kim, Chang Min Park, Myunghee Lee, Sang Joon Park, Yong Sub Song, Jong Hyuk Lee, Eui Jin Hwang, Jin Mo Goo

**Affiliations:** 1 Department of Radiology, Seoul National University College of Medicine, Seoul, Korea; 2 Institute of Radiation Medicine, Seoul National University Medical Research Center, Seoul, Korea; 3 Cancer Research Institute, Seoul National University, Seoul, Korea; Chongqing University, CHINA

## Abstract

**Purpose:**

To identify the impact of reconstruction algorithms on CT radiomic features of pulmonary tumors and to reveal and compare the intra- and inter-reader and inter-reconstruction algorithm variability of each feature.

**Methods:**

Forty-two patients (M:F = 19:23; mean age, 60.43±10.56 years) with 42 pulmonary tumors (22.56±8.51mm) underwent contrast-enhanced CT scans, which were reconstructed with filtered back projection and commercial iterative reconstruction algorithm (level 3 and 5). Two readers independently segmented the whole tumor volume. Fifteen radiomic features were extracted and compared among reconstruction algorithms. Intra- and inter-reader variability and inter-reconstruction algorithm variability were calculated using coefficients of variation (CVs) and then compared.

**Results:**

Among the 15 features, 5 first-order tumor intensity features and 4 gray level co-occurrence matrix (GLCM)-based features showed significant differences (p<0.05) among reconstruction algorithms. As for the variability, effective diameter, sphericity, entropy, and GLCM entropy were the most robust features (CV≤5%). Inter-reader variability was larger than intra-reader or inter-reconstruction algorithm variability in 9 features. However, for entropy, homogeneity, and 4 GLCM-based features, inter-reconstruction algorithm variability was significantly greater than inter-reader variability (p<0.013).

**Conclusions:**

Most of the radiomic features were significantly affected by the reconstruction algorithms. Inter-reconstruction algorithm variability was greater than inter-reader variability for entropy, homogeneity, and GLCM-based features.

## Introduction

Radiomics is the process of extracting quantitative imaging features, including the intra-tumoral heterogeneity, with spatial distribution of pixel values [1]. This method has been investigated in the field of radiology and radiation oncology in various tumors, such as lung cancer, breast cancer, and colorectal cancer. In lung cancer patients, it has been reported that the radiomic features are useful for predicting treatment response [2, 3] and patient survival [4–7].

Assessing the measurement variability is an essential issue for the quantitative data (including radiomic features) as diagnosis and treatment are often guided on the assumption that computed tomographic (CT) measurements are essentially precise and that any measured change reflects a true change in size [8]. However, measured values may vary substantially according to patient factors, image acquisition factors, and radiologist factors [8]. Therefore, identification of the range of variability and the affecting factors are of utmost importance.

Recently, a number of studies investigated the inter-reader and inter-scan variability of radiomic features for the feature selection to reduce dimensionality [4, 9] and focused on the influence of scanning factors (reconstruction kernel and slice thickness) and CT scanners on the measurement variability [10, 11]. In addition, Solomon et al. [12] reported the impact of radiation dose settings and reconstruction algorithms on radiomic feature values with lung nodules of unknown pathology from four patients. However, to the best of our knowledge, analysis of the impact of reconstruction algorithms on radiomic features for oncology patients, and the comparison of inter-reconstruction algorithm variability with the inter-reader variability, have not been performed to date.

Therefore, the aim of the present study was to identify the impact of reconstruction algorithms on CT radiomic features of pulmonary tumors and to reveal the intra- and inter-reader and inter-reconstruction algorithm variability of each feature. We also compared the variability degree, pairwise, to demonstrate the most influential variability factor for the radiomic features.

## Materials and Methods

This retrospective study was approved by the Institutional Review Board of Seoul National University Hospital (IRB No. 1512-016-726) with waivers of informed consent from involved patients as the data were analyzed retrospectively and anonymously.

### Study population

We retrospectively identified 47 oncology patients with pulmonary nodules or masses who underwent contrast-enhanced chest CT on a single CT system (Somatom Definition; Siemens Medical Solutions, Forchheim, Germany) for their clinical indications (i.e., routine follow-up) from September 2013 to October 2013. Among the 47 patients, five were excluded due to the following reasons: (a) patients without a measurable lesion (n = 4) and (b) a patient with ground-glass nodule (n = 1). For the patients with multiple lesions, we chose a dominant measurable lung lesion per patient. Therefore, 42 patients (M:F = 19:23; mean age, 60.43±10.56 years; range, 33–81 years) with 42 lesions (mean size, 22.6±8.5 mm; range, 10.0–41.7 mm) were included in our study.

Among the 42 tumors, there were 8 lung cancers, 5 colon cancers, 5 breast cancers, 4 renal cell carcinomas, 3 rectal cancers, 2 ampulla of Vater cancers, 2 nasopharyngeal cancers, 2 ovarian cancers, and 2 salivary gland cancers. The rest were adenoid cystic carcinoma, cholangiocarcinoma, epithelioid hemangioendothelioma, floor of mouth cancer, gallbladder cancer, hepatocellular carcinoma, melanoma, pancreatic cancer, and retroperitoneal liposarcoma. All patients underwent, or were undergoing, chemotherapy.

### CT acquisition

All CT examinations were performed using a 64-detector row Definition scanner at full inspiration state. Detailed scanning parameters were as follows: 0.6×64 mm detector collimation, 120 kVp, 150 quality-reference mAs, 0.5 sec gantry rotation time, pitch of 1, 512 x 512 matrix, 1.0 mm reconstruction increment and section thickness of 1.0 mm. The image element size (voxel dimension) was 0.68×0.68×1.00 mm. A total of 70–90 mL of 370 mgI/mL of the nonionic contrast material, iopromide (Ultravist 370; Schering, Berlin, Germany), was injected at a rate of 2.3–3.0 mL/sec using a power injector (Stellent Dual; MEDRAD Inc., Warrendale, PA, USA). The CT scans were initiated 60 seconds after the start of the contrast administration. Half-dose images were created using projection data from a single tube of the dual source scans to simulate a situation that requires noise-reducing iterative reconstruction algorithm [13]. Then, the images were reconstructed with filtered back projection (FBP; B50f kernel) and Sinogram Affirmed Iterative Reconstruction (SAFIRE; Siemens Healthcare, Forchheim, Germany; corresponding I50f kernel) at noise reduction strength of level 3 (S3) and 5 (S5), respectively.

For radiation dose assessment, the volume CT dose index (CTDI_vol_) and dose-length product (DLP) for half-dose images were obtained. Estimated effective dose was also calculated from the DLP with conversion factor of 0.0145 from the International Commission on Radiological Protection (ICRP) publication 103 recommendations [14].

### Radiomic feature extraction

Nodule segmentation and analysis were performed by one radiologist (H.K. with 6 years of experience in chest CT) and one CT technician (M.L. with 5 years of research experience in chest CT), independently. One of the readers (H.K.) conducted the overall image analysis twice at an interval of 4 weeks to calculate the intra-reader variability.

**Fig 1:**
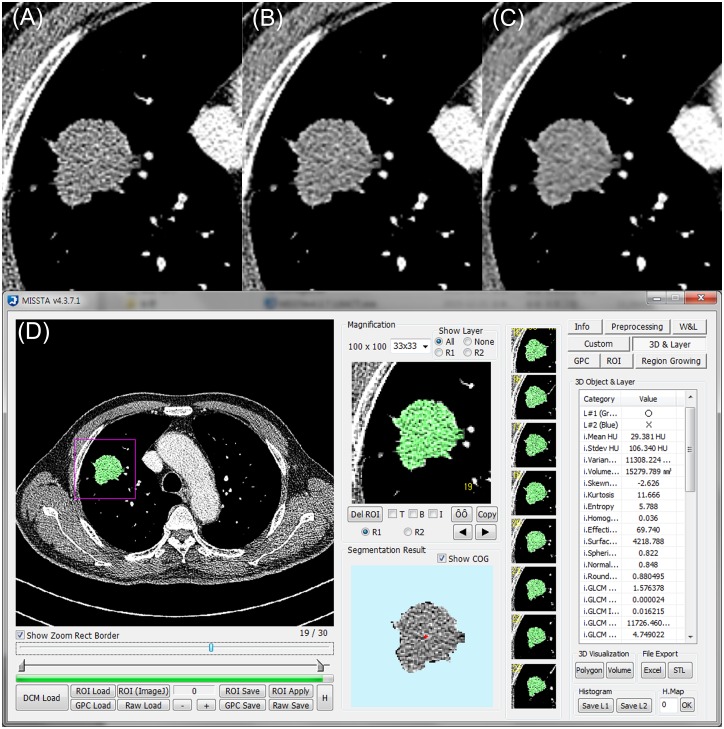
A pulmonary mass (nasopharyngeal carcinoma metastasis) in a 59-year-old male. CT images were reconstructed with (A) filtered back projection and Sinogram Affirmed Iterative Reconstruction (B) level 3 and (C) level 5. (D) Tumor was segmented manually and radiomic features were automatically calculated using in-house software.

Digital imaging and communications in medicine (DICOM) files were transferred from the picture archiving and communication system (PACS) to a personal computer and then loaded to an in-house software program (Medical Imaging Solution for Segmentation and Texture Analysis) [5, 15–17] (Fig 1). This in-house software program was implemented using dedicated C++ language with Microsoft Foundation Classes (Microsoft, Redmond, WA) [5, 15–17]. The tumor boundary was segmented manually on every slice of FBP images to include the entire tumor volume and was saved as a region-of-interest (ROI) file. The ROI file for each case was then copied-and-pasted to the other images reconstructed with S3 and S5. Potential influence of iterative reconstruction algorithm on manual nodule margin delineation was not considered as the contrast between solid lung nodule and background lung parenchyma is intrinsically very high on CT. Further manual editing of ROIs on the images with S3 and S5 was not performed. First-order tumor intensity-based features (mean, standard deviation [SD], skewness, kurtosis, entropy, and homogeneity), size/shape features (volume, effective diameter [ED], surface area [SA], sphericity, and discrete compactness [DC]) and second-order features calculated from the gray level co-occurrence matrix (GLCM) (moments, inverse difference moment [IDM], contrast, and entropy) were automatically obtained at each reconstruction algorithm. In total, we acquired nine datasets of radiomic feature values from three reconstruction algorithms and two readers.

### Statistical analysis

To compare the radiomic feature values among reconstruction algorithms (FBP, S3, and S5), we conducted an analysis of variance (ANOVA) or the Friedman test, as appropriate, after the Shapiro-Wilk test to determine the normality of variables. Subsequent pairwise post-hoc Tukey test or Wilcoxon signed rank test was performed. Analysis was carried out separately with reader 1 and 2 data as there were no significant interactions between the reconstruction algorithm and the reader.

The degree of intra- and inter-reader variability and inter-reconstruction algorithm variability was analyzed using coefficients of variation (CVs). CV was calculated as SD divided by the mean. CVs of intra- and inter-reader variability were calculated based on FBP images. CVs of inter-reconstruction algorithm variability were calculated pairwise (FBP and S3; FBP and S5; S3 and S5) using the data of reader 1. As the exact same ROIs containing the entire tumor volume were applied to the three reconstruction algorithms, the inter-reconstruction algorithm variability consisted solely of the variation due to the change in reconstruction algorithms without the interference of intra- or inter-reader variability. Thereafter, CVs were categorized into four groups; very small (CV≤5%), small (5%<CV≤10%), intermediate (10%<CV≤20%), and large (CV>20%) range of variation [18]. Then, CVs were compared between intra-reader variability and inter-reader variability and between inter-reader variability and inter-reconstruction algorithm variability. CV comparisons were also performed between each pair of inter-reconstruction algorithm variability (FBP and S3 vs. FBP and S5; FBP and S3 vs. S3 and S5; FBP and S5 vs. S3 and S5) to investigate the most influential reconstruction algorithm switch for radiomic feature extraction.

All statistical analyses were performed using SPSS 19.0 (IBM SPSS Statistics, Armonk, NY). A P value of less than 0.05 was indicative of a significant difference and a Bonferroni correction was applied to the multiple comparisons. All data of radiomic features are available in S1 Dataset.

## Results

### Effect of reconstruction algorithm on radiomic features

Radiomic feature values were compared among FBP, S3 and S5 with subsequent post-hoc analysis. Among the 15 features, nine features showed significant differences (p<0.05) among reconstruction algorithms. However, homogeneity and size/shape-based features (volume, ED, SA, sphericity, and DC) did not show significant differences (p>0.05) among reconstruction algorithms. On post-hoc analysis, seven out of nine features demonstrated significant differences according to the level of SAFIRE algorithm (level 3 vs. level 5; p<0.001). The results were concordant between the two readers (Tables 1 and 2).

**Table 1:** Comparison of radiomic feature values between FBP and iterative reconstruction algorithm (Reader 1).

	Median ± SD (range)				Pairwise comparison^a^		
Feature	FBP	S3	S5	Comparison among FBP, S3 and S5^a^	FBP vs. S3	FBP vs. S5	S3 vs. S5
Mean (HU)	28.26 ± 45.34 (-168.48, 102.33)	29.85 ± 45.52 (-169.06, 103.33)	28.55 ± 45.80 (-171.17, 102.74)	<0.001	<0.001	0.621	<0.001
SD (HU)	110.36 ± 41.90 (54.80 ± 229.67)	97.35 ± 44.06 (42.97, 229.42)	90.42 ± 46.55 (32.40, 227.00)	<0.001	<0.001	<0.001	<0.001
Skewness	-1.52 ± 0.80 (-2.75, 0.13)	-2.11 ± 0.94 (-3.46, 0.13)	-2.55 ± 1.05 (-4.15, 0.15)	<0.001	0.069	0.000	0.055
Kurtosis	4.67 ± 4.11 (0.17, 14.42)	7.10 ± 5.75 (0.26, 21.37)	12.25 ± 7.79 (0.80, 29.06	<0.001	<0.001	<0.001	<0.001
Entropy	5.81 ± 0.25 (5.41, 6.58)	5.63 ± 0.27 (5.17, 6.53)	5.44 ± 0.31 (4.88, 6.48)	<0.001	<0.001	<0.001	<0.001
Homogeneity	0.033 ± 0.005 (0.024, 0.042)	0.035 ± 0.007 (0.020, 0.048)	0.037 ± 0.011 (0.014, 0.060)	0.113			
Volume (mm^3^)	2990.02 ± 5244.62 (208.88, 23351.47)	2990.02 ± 5244.62 (208.88, 23351.47)	2990.02 ± 5244.62 (208.88, 23351.47)	N/A^b^			
ED (mm)	30.84 ± 18.32 (8.15, 86.22)	30.84 ± 18.32 (8.15, 86.22)	30.84 ± 18.32 (8.15, 86.22)	N/A^b^			
SA (mm^2^)	1418.49 ± 2276. 32 (164.02, 12611.04)	1418.49 ± 2276. 32 (164.02, 12611.04)	1418.49 ± 2276. 32 (164.02, 12611.04)	N/A^b^			
Sphericity	0.84 ± 0.13 (0.33, 0.97)	0.84 ± 0.13 (0.33, 0.97)	0.84 ± 0.13 (0.33, 0.97)	N/A^b^			
DC	0.80 ± 0.17 (0.23, 1.00)	0.80 ± 0.17 (0.23, 1.00)	0.80 ± 0.17 (0.23, 1.00)	N/A^b^			
GLCM moments	1.74 ± 0.25 (1.20, 2.10)	1.50 ± 0.31 (0.97, 2.06)	1.20 ± 0.34 (0.72, 2.11)	<0.001	0.005	<0.001	<0.001
GLCM IDM	0.015 ± 0.004 (0.007, 0.024)	0.021 ± 0.005 (0.008, 0.033)	0.033 ± 0.009 (0.014, 0.051)	<0.001	<0.001	<0.001	<0.001
GLCM contrast	15062.98 ± 8327.51 (4452.02, 40548.48)	12432.71 ± 8286.55 (2528.05, 38082.93)	10550.86 ± 8051.43 (1360.77, 35679.29)	<0.001	<0.001	<0.001	<0.001
GLCM entropy	4.41 ± 0.34 (3.40, 5.06)	4.32 ± 0.30 (3.40, 4.88)	4.18 ± 0.26 (3.39, 4.64)	0.009	0.454	0.006	0.140

DC, discrete compactness; ED, effective diameter; FBP, filtered back projection; GLCM, gray level co-occurrence matrix; HU, Hounsfield unit; IDM, inverse difference moment; N/A, not available; SA, surface area; SD, standard deviation; S3, Sinogram Affirmed Iterative Reconstruction level 3; S5, Sinogram Affirmed Iterative Reconstruction level 5

^a^ Data are p values for each comparison.

^b^ P value was not available from the Friedman test as the feature values were exactly same in all three groups.

**Table 2:** Comparison of radiomic feature values between FBP and iterative reconstruction algorithm (Reader 2).

	Median ± SD (range)				Pairwise comparison^a^		
Feature	FBP	S3	S5	Comparison among FBP, S3, and S5^a^	FBP vs. S3	FBP vs. S5	S3 vs. S5
Mean (HU)	35.05 ± 38.55 (-137.85, 104.72)	37.58 ± 38.67 (-137.77, 105.38)	36.05 ± 38.88 (-139.66, 104.53)	<0.001	<0.001	0.420	<0.001
SD (HU)	91.71 ± 32.94 (56.73, 224.60)	80.52 ± 34.57 (45.57, 224.31)	72.10 ± 36.53 (35.55, 220.94)	<0.001	<0.001	<0.001	<0.001
Skewness	-1.28 ± 1.01 (-4.34, 1.04)	-1.48 ± 1.11 (-4.82, 0.19)	-2.08 ± 1.37 (-5.32, 1.22)	0.004	0.193	0.003	0.228
Kurtosis	3.12 ± 6.13 (-0.24, 27.35)	5.20 ± 7.68 (-0.27, 31.92)	8.23 ± 9.79 (-0.31, 37.48)	<0.001	<0.001	<0.001	<0.001
Entropy	5.74 ± 0.24 (5.43, 6.47)	5.57 ± 0.25 (5.20, 6.41)	5.36 ± 0.29 (4.92, 6.35)	<0.001	<0.001	<0.001	<0.001
Homogeneity	0.033 ± 0.005 (0.023, 0.042)	0.035 ± 0.007 (0.019, 0.048)	0.038 ± 0.012 (0.014, 0.059)	0.089			
Volume (mm^3^)	3223.20 ± 5262.79 (207.01, 25082.82)	3223.20 ± 5262.79 (207.01, 25082.82)	3223.20 ± 5262.79 (207.01, 25082.82)	N/A^b^			
ED (mm)	32.03 ± 18.40 (8.12, 89.35)	32.03 ± 18.40 (8.12, 89.35)	32.03 ± 18.40 (8.12, 89.35)	N/A^b^			
SA (mm^2^)	1245.36 ± 1968.70 (149.54, 10062.38)	1245.36 ± 1968.70 (149.54, 10062.38)	1245.36 ± 1968.70 (149.54, 10062.38)	N/A^b^			
Sphericity	0.87 ± 0.13 (0.38, 1.00)	0.87 ± 0.13 (0.38, 1.00)	0.87 ± 0.13 (0.38, 1.00)	N/A^b^			
DC	0.86 ± 0.15 (0.33, 1.12)	0.86 ± 0.15 (0.33, 1.12)	0.86 ± 0.15 (0.33, 1.12)	N/A^b^			
GLCM moments	1.72 ± 0.25 (1.23, 2.14)	1.50 ± 0.30 (1.05, 2.08)	1.17 ± 0.34 (0.71, 2.10)	<0.001	<0.001	<0.001	<0.001
GLCM IDM	0.015 ± 0.004 (0.007, 0.023)	0.022 ± 0.005 (0.009, 0.032)	0.034 ± 0.009 (0.014, 0.048)	<0.001	<0.001	<0.001	<0.001
GLCM contrast	13984.14 ± 7300.42 (5561.31, 39429.17)	11332.52 ± 7034.59 (3691.25, 36268.55)	9761.97 ± 6746.66 (1820.66, 33914.11)	<0.001	<0.001	<0.001	<0.001
GLCM entropy	4.41 ± 0.35 (3.39, 5.05)	4.32 ± 0.30 (3.39, 4.86)	4.17 ± 0.25 (3.38, 4.58)	0.008	0.446	0.006	0.134

DC, discrete compactness; ED, effective diameter; FBP, filtered back projection; GLCM, gray level co-occurrence matrix; HU, Hounsfield unit; IDM, inverse difference moment; N/A, not available; SA, surface area; SD, standard deviation; S3, Sinogram Affirmed Iterative Reconstruction level 3; S5, Sinogram Affirmed Iterative Reconstruction level 5

^a^ Data are p values for each comparison.

^b^ P value was not available from the Friedman test as the feature values were exactly same in all three groups.

### Comparison of variability using CV

As for the variability of radiomic features (which is attributable to either inter-reader or inter-reconstruction algorithm variation), ED, sphericity, entropy, and GLCM entropy exhibited a very small variation (CV≤5%). SA, volume, and DC showed a small variation (5%<CV≤10%) and homogeneity and SD showed intermediate variability (10%<CV≤20%). Other features of GLCM moments, GLCM contrast, GLCM IDM, kurtosis, skewness, and mean demonstrated a wide range of variation (CV>20%). Detailed data are displayed in Table 3.

**Table 3:** Coefficient of variation (CV) of radiomic features.

Feature	Intra-reader CV	Inter-reader CV	CV of FBP/S3	CV of FBP/S5	CV of S3/S5
Mean	50.3	131.3	25.4	8.3	13.1
SD	11.5	15.1	6.8	13.4	6.7
Skewness	26.2	94.5	23.3	42.1	22.9
Kurtosis	27.7	62.1	31.4	52.2	25.6
Entropy	1.1	1.3	2.1	4.6	2.5
Homogeneity	1.0	1.6	7.8	15.0	8.6
Volume^a^	5.1	5.4			
ED^a^	2.5	2.7			
SA^a^	4.1	5.3			
Sphericity^a^	3.6	4.8			
DC^a^	5.7	6.7			
GLCM moments	1.2	1.4	9.9	24.5	14.9
GLCM IDM	3.6	3.3	25.0	53.3	30.4
GLCM contrast	11.3	12.7	15.5	30.0	15.5
GLCM entropy	0.6	0.6	1.3	3.3	2.1

Data are CV in percentage (%).

DC, discrete compactness; ED, effective diameter; FBP, filtered back projection; GLCM, gray level co-occurrence matrix; IDM, inverse difference moment; SA, surface area; SD, standard deviation; S3, Sinogram Affirmed Iterative Reconstruction level 3; S5, Sinogram Affirmed Iterative Reconstruction level 5

^a^ Inter-reconstruction algorithm variability of size/shape features were zero as the same ROI were copied-and-pasted from FBP images to S3 and S5.

Inter-reader variability of radiomic features was larger than intra-reader variability except for that of GLCM IDM, although the statistical significance between inter- and intra-reader variability was found only for skewness and kurtosis (p<0.001) (S1 Table). Inter-reader variability was also larger than inter-reconstruction algorithm variability for nine out of 15 features. However, for entropy, homogeneity, GLCM moments, GLCM IDM, GLCM contrast, and GLCM entropy, inter-reconstruction algorithm variability was significantly greater than inter-reader variability (p<0.013). Inter-reconstruction algorithm variability for the first-order tumor intensity features and GLCM-based features was largest between FBP and S5 (p<0.017), except for the mean (FBP and S3) (S2 Table).

### Radiation dose

Mean CTDI_vol_ and DLP of the CT scans were 5.21±1.69 mGy (range, 1.06–10.14 mGy) and 194.68±63.84 mGy∙cm (range, 46.00–351.50 mGy∙cm), respectively. Mean effective dose was 2.82±0.93 mSv (range, 0.67–5.10 mSv).

## Discussion

In this study, we have identified that the impact of a reconstruction algorithm was significant on most of the first-order tumor intensity features (5/6) and second-order GLCM-based features (4/4). Homogeneity and size/shape features were not influenced by the reconstruction algorithm in both readers. With regard to the measurement variability, ED, sphericity, entropy, and GLCM entropy were the most robust features (CV≤5%). Inter-reader variability was the largest contributing variation for first-order features (9/11). However, for entropy, homogeneity and four other GLCM-based features, inter-reconstruction algorithm variability was significantly larger than inter-reader variability. For the pairwise inter-reconstruction algorithm variability comparisons, variation between FBP and S5 was largest for the first-order tumor intensity features (5/6) and GLCM-based features (4/4).

Recently, Solomon et al. [12] reported similar results about the radiation dose settings and reconstruction algorithms significantly affecting the radiomic feature values of liver lesions, lung nodules, and kidney stones in 20 patients. In that study, adaptive statistical iterative reconstruction (ASIR) had a significant effect on one of the features (SD) and model-based iterative reconstruction (MBIR) had a significant effect on 11 quantitative features (volume, sphericity, attenuation, background noise, contrast-to-noise ratio, in-plane blur, axial blur, SD, skewness, GLCM contrast, and GLCM IDM) for lung nodules. Excluding the image quality metrics, five first-order based features and two GLCM-based features were affected by MBIR. However, Solomon et al. [12] dealt with only nine lung nodules from four patients for whom the final diagnoses were not disclosed and the intra- and inter-reader variability in their measurements were not analyzed. Comprehensive investigation into the inherent variability of measurement (including the radiologist factor) is critical given that the major contributing factor, which might be either the radiologist factor or image acquisition factor, determines the total variation (regardless of other minor factors).

In the present study, most of the radiomic features (excluding size/shape features) were influenced by the reconstruction algorithms in contrast to the study by Solomon et al. [12], which reported that less than half of the pixel value distribution features and GLCM-based features were affected. The discrepancy in results for the size/shape features is due to different segmentation methods. We performed nodule segmentation manually on FBP images and the segmentation profile, which were saved as ROI files, were then pasted to the other reconstruction images without further correction. This approach was adopted to analyze the effect of the reconstruction algorithm without any interference from the variability related to the nodule segmentation. In contrast, Solomon et al. [12] conducted semi-automated segmentation at each algorithm setting, which induced intra-reader variability of semi-automatic segmentation plus inter-reconstruction algorithm variation. In addition, entropy and GLCM entropy were not influenced by the reconstruction algorithms in that study [12], whereas both of them showed significant decreases on the iterative reconstruction in our results. Entropy is one of the most representative metrics of tumor heterogeneity and has been reported to have significant association with patient survival in non-small cell lung cancer patients [5, 7]. Given that entropy is a measure of image irregularity [5] and iterative reconstruction algorithm directly reduces noise and artifacts resulting from irregularities (such as photon starvation, beam hardening, and nonlinearity of individual detector elements) [19], it is plausible that entropy decreases when the iterative reconstruction algorithm is applied. Therefore, it has to be noted that different entropy cutoff levels should be used when analyzing CT images of cancer patients with various reconstruction algorithms and that the change of entropy value in patients should be carefully evaluated if different reconstruction algorithms were used. The discordant results between this study and the one by Solomon et al. [12] might be explained by the different study population (e.g., nodule size or enhancement), study sample size, and iterative reconstruction algorithms (SAFIRE vs. ASIR and MBIR) used. Variation of radiomic features according to the different iterative reconstruction algorithms warrants further investigation.

In addition to the impact of reconstruction algorithm itself, SAFIRE noise reduction level was also an influencing factor to the radiomic features. Values of four out of six first-order tumor intensity features and three out of four GLCM-based features were significantly different between S3 and S5. When the pairwise CV comparisons of inter-reconstruction algorithm variability were performed, the variability between FBP and S5 was significantly higher than other inter-reconstruction algorithm variabilities, except for the mean. In other words, the inter-reconstruction algorithm variability became greater as the degree of noise reduction between the two CT images increased.

Another notable finding in our study was that the inter-reconstruction algorithm variability for entropy, homogeneity, and 4 GLCM-based features was significantly larger than the inter-reader variability. This is an interesting result considering that conventional volumetric variability studies revealed that nodule volumetry was robust to the reconstruction algorithm and the measurement variability was primarily attributed to the inter-reader or inter-scan variability [20–23]. Volume measurement itself is not comparable to the radiomic analysis. However, radiologists should be aware of the fact that the alteration in the reconstruction method can become a dominant source of measurement variability and its effect can be even greater than the change among readers for certain radiomic features, particularly texture features.

There were several limitations in our study other than the intrinsic limits of any retrospective study. First, the levels of SAFIRE noise reduction strength were chosen empirically. The level refers to the amount of noise reduction that is desired in the image and is not related to the number of iterations [24]. We selected level 3 and 5 to get the average, and highest, noise reduction. Second, radiomic features obtained with gray level run length matrices, or Laplacian of Gaussian filter methods, were not tested in the present study. Therefore, our study results cannot be applied to the features obtained through those methods. Third, our study results might be reconstruction-algorithm specific. Investigations into the effects of various iterative reconstruction algorithms on radiomic features are warranted in the future. Fourth, the biological ranges of the radiomic features in patients with disease improvement, or progression, were not investigated. Thus, the transition and range of radiomic feature values in various clinical situations should be studied to reveal the clinical significance of the measurement variability. Fifth, the study population comprised heterogeneous group of tumor histology. However, the focus of our study was not on the absolute or true value of each feature, rather it was on the change and variability of features according to the transition in reconstruction algorithm while other scanning parameters were fixed. The effect of tumor histology on variability analysis of the present study was considered to be minimal.

In conclusion, most of the radiomic features extracted from pulmonary oncology patients were significantly affected by the reconstruction algorithms. Inter-reconstruction algorithm variability was the major contributing variation for entropy, homogeneity, and GLCM-based features, while inter-reader variability was more significant in many first-order features.

## Supporting Information

S1 Dataset Radiomic feature data of the study population. (XLSX)

S1 Table Coefficient of variation comparison between inter-reader variability and intra-reader variability and between inter-reader variability and inter-reconstruction algorithm variability. (DOC)

S2 Table Pairwise coefficient of variation comparisons among inter-reconstruction algorithm variability. (DOC)
