# Supplementary material for: Impact of Reconstruction Algorithms on CT Radiomic Features of Pulmonary Tumors: Analysis of Intra- and Inter-Reader Variability and Inter-Reconstruction Algorithm Variability
Source: PLoS One. 2016 Oct 14;11(10):e0164924. doi: 10.1371/journal.pone.0164924 (PMC5065199; doi:10.1371/journal.pone.0164924)
Supplement: S1 Table — (DOC) [file pone.0164924.s002.doc]

**S1 Table. CV comparison between inter-reader variability and intra-reader variability and between inter-reader variability and inter-reconstruction algorithm (Inter-RA) variability.**

|  |  | **Inter-reader variability vs. Inter-RA variability** | | |
| --- | --- | --- | --- | --- |
| **Feature** | **Inter- vs. Intra-reader variability** | **Inter-reader variability vs. FBP/S3 variability** | **Inter-reader variability vs. FBP/S5 variability** | **Inter-reader variability vs. S3/S5 variability** |
| Mean | 0.352 | <0.001 | <0.001 | <0.001 |
| SD | 0.315 | 0.002 | 0.553 | 0.001 |
| Skewness | <0.001 | <0.001 | 0.083 | <0.001 |
| Kurtosis | <0.001 | <0.001 | 0.365 | <0.001 |
| Entropy | 0.472 | 0.002 | 0.000 | 0.000 |
| Homogeneity | 0.080 | <0.001 | <0.001 | <0.001 |
| Volume | 0.778 | <0.001 | <0.001 | <0.001 |
| ED | 0.778 | <0.001 | <0.001 | <0.001 |
| SA | 0.093 | <0.001 | <0.001 | <0.001 |
| Sphericity | 0.027 | <0.001 | <0.001 | <0.001 |
| DC | 0.037 | <0.001 | <0.001 | <0.001 |
| GLCM moments | 0.364 | <0.001 | <0.001 | <0.001 |
| GLCM IDM | 0.494 | <0.001 | <0.001 | <0.001 |
| GLCM contrast | 0.759 | 0.163 | <0.001 | 0.138 |
| GLCM entropy | 0.759 | <0.001 | <0.001 | <0.001 |

Note.***—***Data are p-values for each comparison.

CV, coefficient of variation; DC, discrete compactness; ED, effective diameter; FBP, filtered back projection; GLCM, gray level co-occurrence matrix; IDM, inverse difference moment; SA, surface area; SD, standard deviation; S3, Sinogram Affirmed Iterative Reconstruction level 3; S5, Sinogram Affirmed Iterative Reconstruction level 5
