# Supplementary material for: Impact of Reconstruction Algorithms on CT Radiomic Features of Pulmonary Tumors: Analysis of Intra- and Inter-Reader Variability and Inter-Reconstruction Algorithm Variability
Source: PLoS One. 2016 Oct 14;11(10):e0164924. doi: 10.1371/journal.pone.0164924 (PMC5065199; doi:10.1371/journal.pone.0164924)
Supplement: S2 Table — (DOC) [file pone.0164924.s003.doc]

**S2 Table. Pairwise CV comparisons among inter-reconstruction algorithm variability.**

| **Feature** | **FBP/S3 variability vs. FBP/S5 variability** | **FBP/S3 variability vs. S3/S5 variability** | **FBP/S5 variability vs. S3/S5 variability** |
| --- | --- | --- | --- |
| Mean | 0.006 | 0.449 | 0.005 |
| SD | <0.001 | 0.218 | <0.001 |
| Skewness | <0.001 | <0.001 | <0.001 |
| Kurtosis | <0.001 | <0.001 | <0.001 |
| Entropy | <0.001 | 0.173 | <0.001 |
| Homogeneity | <0.001 | 0.864 | <0.001 |
| Volume | 1.000 | 1.000 | 1.000 |
| ED | 1.000 | 1.000 | 1.000 |
| SA | 1.000 | 1.000 | 1.000 |
| Sphericity | 1.000 | 1.000 | 1.000 |
| DC | 1.000 | 1.000 | 1.000 |
| GLCM moments | <0.001 | 0.014 | <0.001 |
| GLCM IDM | <0.001 | 0.001 | <0.001 |
| GLCM contrast | <0.001 | 0.827 | <0.001 |
| GLCM entropy | <0.001 | 0.024 | <0.001 |

Note.***—***Data are p-values for each comparison.

CV, coefficient of variation; DC, discrete compactness; ED, effective diameter; FBP, filtered back projection; GLCM, gray level co-occurrence matrix; IDM, inverse difference moment; SA, surface area; SD, standard deviation; S3, Sinogram Affirmed Iterative Reconstruction level 3; S5, Sinogram Affirmed Iterative Reconstruction level 5
